# Improving Cognition in Older Adults in Community Housing (ICOACH)

**DOI:** 10.1002/alz70860_102362

**Published:** 2025-12-23

**Authors:** Bernadette Mdawar, Christopher R Bowie, Tarek K Rajji, Angela C Golas

**Affiliations:** ^1^ Centre for Addiction and Mental Health, Toronto, ON, Canada; ^2^ Temerty Faculty of Medicine, Division of Psychiatry, University of Toronto, Toronto, ON, Canada; ^3^ Queen's University, Kingston, ON, Canada; ^4^ UT Southwestern University, Dallas, TX, USA; ^5^ University of Toronto, Toronto, ON, Canada

## Abstract

**Background:**

Mental health conditions, often associated with cognitive impairment, significantly contribute to seniors' vulnerabilities. This, in turn, reinforces the misconception that the elderly are a burden to society, further intensifying social isolation and physical disability. Growing evidence supports the use of multimodal interventions as both a preventative and therapeutic tool for seniors with mental health conditions. Our goal is to design an integrated intervention that is both feasible and tolerable, for older adult with mental illness living in senior community housing.

**Method:**

The ICOACH is an open‐label study of a 12‐week integrated program combining computerized cognitive remediation (CCR) with social and physical activities. The program's activities were iteratively modified based on qualitative analysis of participants' feedback, which was gathered through semi‐structured interviews, as well as their responses to the Satisfaction and System Usability Scale (SUS).

**Result:**

Of the fifteen enrolled participants from 3 different community housing locations, eleven completed the full program. Reasons for withdrawal included relocating to a new residence, conflicts with other participants, and a loss of interest in the program. Baseline characteristics were for group 1 (*n* = 5; mean age[SD] years=75 [7.2]; mean MoCA [SD] = 20.6 [4.3]), group 2 (*n* = 6; mean age[SD] years=69.3 [7.2]; mean MoCA [SD] = 22 [3.8]) and group 3 (*n* = 4; mean age[SD] years=71 [10]; mean MoCA [SD] = 18.5 [4.2]). All participants were highly satisfied with the program, notably 72.7% of participants agreed or somewhat agreed that the program had improved their quality of life in their housing. There was an upward trend in the usability of the CCR component, with each successive group showing improved usability. Attendance for social activities was the highest across all groups, followed by CCR and then physical activities. Conflicting participants’ medical and social appointments and site‐related logistical issues were main external barriers for attendance. Internal barriers included reported cognitive difficulties and low motivation. The role of the interventionist and break time were highly regarded as key facilitating factors.

**Conclusion:**

Our results showed that an integrated program is well tolerated among older adults with mental health conditions living in community housing, and highlighted key barriers for program implementation in this setting.

